# Exploring the Diagnostic Performance of Magnetic Resonance Imaging in Ultrasound-Guided High-Intensity Focused Ultrasound Ablation for Abdominal Wall Endometriosis

**DOI:** 10.3389/fphys.2022.819259

**Published:** 2022-02-15

**Authors:** Shangying Hu, Yuhang Liu, Rongsheng Chen, Zhibo Xiao

**Affiliations:** ^1^Department of Gynecology and Obstetrics, The University-Town Hospital of Chongqing Medical University, Chongqing, China; ^2^Department of Radiology, The First Affiliated Hospital of Chongqing Medical University, Chongqing, China

**Keywords:** abdominal wall endometriosis, magnetic resonance imaging, high-intensity focused ultrasound, ablation, diagnostic performance

## Abstract

**Objective:**

This study aimed to explore the clinical value of magnetic resonance imaging (MRI) combined with ultrasound-guided high-intensity focused ultrasound (USg-HIFU) for the diagnosis and treatment of abdominal wall endometriosis (AWE).

**Methods:**

Magnetic resonance imaging was performed before and after USg-HIFU. Information on clinical characteristics of patients, MRI characteristics of lesions, and treatment outcomes were collected. Thirty AWE lesions in 29 patients were examined before HIFU treatment, while 27 patients were examined after treatment. The results of MRI and color doppler ultrasound before surgery, as well as the volume and the apparent diffusion coefficient (ADC) values of the lesions before and after USg-HIFU treatment were compared. We also observed the clinical symptoms remission, recurrence, and ablation rates of the lesions in follow-up after HIFU treatment.

**Results:**

The locations of the 30 AWE lesions were identified by MRI before USg-HIFU treatment. Their sizes appeared larger on MRI than ultrasound (*P* < 0.05). A total of 27 lesions were evaluated by MRI after USg-HIFU treatment, of which 92.6% (25/27) lesions were of high or slightly high signal intensity on T1-weighted images, and 77.8% (21/27) lesions were of mixed signal intensity on T2-weighted images. The mean ADC values of AWE lesions were 1.47 (1.20–1.59) × 10^–3^mm^2^/s and 1.86 (1.61–2.12) × 10^–3^mm^2^/s for pre-and post-HIFU treatment (*P* < 0.05). Patients with higher ablation rates (>50%) had a higher complete/partial remission rate than those with lower ablation rates (<50%), and had a lower recurrence rate (*P* < 0.05).

**Conclusion:**

MRI is a useful tool for identifying the location, size, and concurrent changes of AWE before and after USg-HIFU treatment, which is beneficial for follow-up monitoring and defining treatment efficacy.

## Introduction

Endometriosis (EM) occurs when active endometrial tissue proliferates outside the endometrial area (Horton et al., 2008; Neha and Arulselvi, 2010; Zondervan et al., 2020). It is a common gynecological disease among women of reproductive age (Zondervan et al., 2020). Most EM lesions are found in the pelvic cavity (Allen et al., 2021), but abdominal wall EM (AWE) may also develop in association with a previous surgical scar after a cesarean section, abdominal hysterectomy, ectopic pregnancy, and other gynecological surgeries (Horton et al., 2008; Efremidou et al., 2012; Matalliotakis et al., 2020).

Treatment for AWE is mainly focused on controlling symptoms and preventing the deterioration of the lesions (Ecker et al., 2014; Allen et al., 2021). Medications used in the treatment of AWE are not effective and pose serious adverse effects (Gunes et al., 2005; Rindos and Mansuria, 2017). In general, AWE treatment involves local lesion resection. As with other surgeries, there are associated risks such as relapse, post-operative incision hernia, and scarring of the abdominal wall (Evsen et al., 2011; Liu et al., 2013; Rindos and Mansuria, 2017). High-intensity focused ultrasound (HIFU) ablation is a conformal thermal ablation technique that causes coagulation necrosis of target tissues, without damaging surrounding tissues and those in the acoustic pathway (Liu et al., 2018; Zhao et al., 2018). USg-HIFU treatment, with real-time ultrasound imaging guidance, uses alternating compression and rarefaction of sound waves to achieve a therapeutic benefit, and is based on the simple principle of focusing energy waves at a target point to produce a thermal effect (Duc and Keserci, 2019). In the last two decades, HIFU, which is a novel and alternative therapeutic option to conventional therapies, has been performed to treat many diseases of different solid organs such as the brain, thyroid, liver, kidney, pancreas, breast, uterus, prostate, and bone (Duc and Keserci, 2019). Previous studies have shown that HIFU treatment for AWE is safe and effective (Wang et al., 2011; Nguyen, 2020). As a non-invasive treatment, HIFU does not cause scarring, and thus, offers a new and effective therapeutic option for patients with AWE (Luo et al., 2017; Zhao et al., 2018).

Before the availability of HIFU treatment for AWE, ultrasound, puncture biopsy under ultrasound guidance, computed tomography (CT), and magnetic resonance imaging (MRI) were used to detect AWE. Among them, ultrasound is the most convenient method (Allen et al., 2021; Cocco et al., 2021). However, the resolution and target specificity of ultrasound is limited; thus, it cannot provide a precise and reliable diagnosis of lesions (Busard et al., 2010). While puncture biopsy under ultrasound guidance can offer a specific diagnosis, supporting evidence is still limited due to the small sample size evaluated to date, as well as the potential for inducing new metastases (Gupta, 2008; Guerriero et al., 2020). CT is rarely used in the clinical diagnosis of AWE, because it lacks effective contrast for soft tissues and due to concerns regarding radiation exposure (Genç et al., 2014). The use of MRI offers several advantages. One is that there is no need for invasive procedures or radiation exposure (Stein et al., 2012). MRI offers arbitrary azimuthal imaging that clearly detects the edges of lesions and the depth of infiltration (Liu et al., 2018; Allen et al., 2021). For those patients with AWE outside of the pelvic cavity, MRI can identify tiny hemorrhagic lesions (Busard et al., 2010). However, studies describing MRI in AWE are still lacking. The purpose of this study was to explore the feasibility of applying MRI in HIFU treatment for AWE.

## Materials and Methods

### Study Design and Patient Enrollment

This prospective study was approved by the institutional review board. Women diagnosed with AWE by clinical examination (Table 1) or imaging were admitted to the Haifu Center of the First Affiliated Hospital of Chongqing Medical University and Chongqing Haifu Hospital from January 2011 to December 2014. They consented to receive HIFU treatment. MRI examinations were performed before and after HIFU treatment.

**TABLE 1:** The diagnostic criteria of HIFU treatment for AWE.

	Diagnostic criteria
1	Woman of child-bearing age.
2	Previous abdominal surgery, especially cesarean section, abdominal hysterectomy, or surgery for ectopic pregnancy.
3	Neoplasm in incision site, neoplasm with menstruation period pain or tenderness
4	MRI suggesting localized hemorrhage in the surgical scar area or the abdominal wall near scar area.
5	Period pain disappeared after HIFU treatment.
6	Proven to be another diagnosis by surgical pathology examination or fine-needle aspiration biopsy.

### Magnetic Resonance Imaging Protocol

Magnetic resonance imaging was performed on a GE 3.0T MR scanner (Signa HD Excite, General Electric Company, Chicago, IL, United States) before and 1–2 days after HIFU treatment. Standard T1-weighted imaging (T1WI) [repetition time/echo time (TR/TE): 600/10 ms, slice thickness: 6 mm, matrix size: 180 × 384 mm, NEX: 1]; T2WI (TR/TE: 3280/105 ms, slice thickness: 6 mm, matrix size: 256 × 288 mm, NEX: 2); and enhanced-weighted with LAVA sequence (TR/TE: 3.9/1.8, slice thickness: 2 mm, matrix size: 390 × 312 mm), were performed imaging. The pre- and post-surgical imaging acquisition protocols included an axial localization sequence and a T2-weighted sequence followed by a contrast-enhanced T1-weighted series. Gd-DTPA was used as the contrast agent at a dose of 0.1 mmol/kg and was administered intravenously. The apparent diffusion coefficient (ADC) values and the intensity of the diffusion-weighted imaging (DWI) signal of AWE before and after HIFU were measured using the DWI sequence of *b* = 800 s/mm^2^ with Functool software (AW 4.6, GE Healthcare, General Electric Company, Chicago, IL, United States). The central slice that could manifest the largest part of AWE was selected and the regions of interest were placed to contain as much of the AWE tissue as possible at that level. Three independent measurements were taken and the average ADC value was calculated.

### Ultrasound Ablation

Treatment was performed with an USg-HIFU tumor therapeutic system (Model-JC Focused Ultrasound Tumor Therapeutic System, Chongqing Haifu Medical Technology Co., Ltd., Chongqing, China). The treatment parameters were as follows: transducer frequency 0.8 MHz; 200 mm diameter; 145 mm focal distance; 8 mm macro-axis of the focal region; and 3 mm minor axis of the focal region.

The patients were placed in a prone position on the HIFU table, with the anterior abdominal wall in contact with degassed water. A degassed water balloon was placed between the abdominal wall and the transducer to help compress and push the bowel away from the acoustic pathway.

### Imaging Analysis

Pelvic MRI results were reviewed by two experienced radiologists. To measure the size of the AWE before and after ablation, three diameters—the long diameter (D1), anteroposterior diameter (D2), and left-ring diameter (D3)—were considered. The formula used to calculate the ellipse (V) was: *V* = 0.5233 × D1 × D2 × D3. The 3-dimensional (3D) diameter of the non-perfusion areas were either measured or calculated.

### Statistical Analysis

All results were analyzed using SPSS 26.0. The non-parametric Wilcoxon signed-rank test was used to compare the following two sets of data: (1) The maximal radial line and volume of AWE lesions in color doppler ultrasound and pelvic MRI before surgery; (2) The ADC values of the DWI sequence before and after surgery. The Fisher’s exact test was used to determine the statistical significance between the following: ablation rate and symptomatic relief, ablation rate and relapse, and relief and relapse. Statistical significance was defined by *P* < 0.05.

## Results

### Patient Survey

In this study, the median age of the 29 included patients was 32 years [range: 24–42 years, interquartile range (IQR): 30.0–35.8 years]. The number of pregnancies was 2 (IQR: 1–3), while the number of cesarean sections was 1 (IQR: 1–2). The time before abdominal period pain or when the abdominal mass was found varied from 3 to 144 months, and the median period was 36 months (IQR: 24–48 months).

All patients had a history of cesarean section 100% (29/29) and 89.7% (26/29) cases had a transverse incision. Additionally, 96.6% (28/29) attended the clinic with a mass and period pain in the scar area. More detailed data are presented in Table 2.

**TABLE 2:** Baseline characteristics.

Characteristic	Number of cases	Percentage
**History of cesarean section**		
–A single cesarean section	28	96.5
–Repeated cesarean sections (≥2 times)	1	3.45
**Associated with other surgical history**		
–Laparoscopic surgical chocolate cyst removal	1	3.45
**Location of cesarean section incision**		
–Transverse incision	26	89.7
–Vertical incision	3	10.3
**Self-reported upon doctor visit**		
–A mass and period pain in the scar area	28	96.5
–Asymptomatic	1	3.45
**Number of lesions on palpation**		
–Single lesion	28	96.5
–Multiple lesions (≥2)	1	3.45
**Other physical signs**		
–Enlarged uterus	5	17.2
–Cyst masses in adnexal area	5	17.2
–Nodules in posterior vaginal fornix	1	3.45

### Color Flow Doppler Ultrasonography Examinations

**FIGURE 1:**
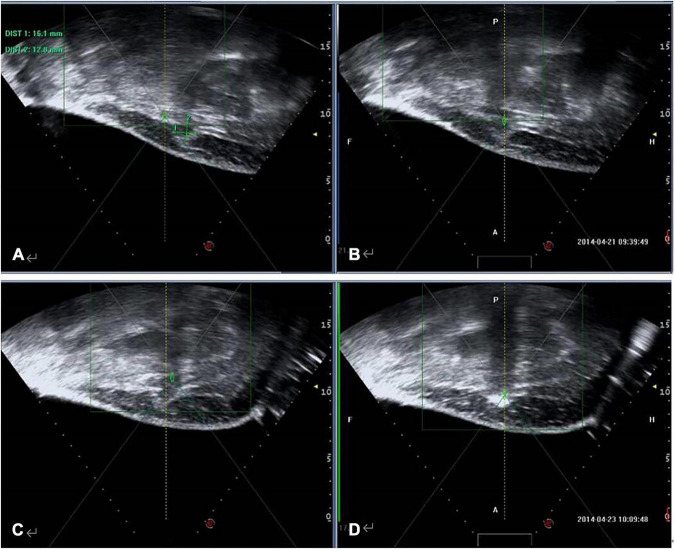
Ultrasound imaging in a 35-year-old woman with AWE. Intraoperative ultrasound was used to measure and locate the AWE lesion. **(A)** Before HIFU ablation, sagittal-guided ultrasound showed a low echogenic nodule of approximately 16.1 × 12.8 mm in the right subcutaneous muscle layer of the abdominal wall incision (white arrow). **(B)** Focused ultrasound focus localization was displayed before ablation (white arrow). **(C)** During the operation, ultrasound energy of fixed-point emission (white arrow). **(D)** The focus position showed a massive hyperechoic change (white arrow), but there was no significant echo change in the superficial tissue of the lesion.

A gynecological ultrasound was performed before surgery for all patients; intraoperative ultrasound was used to measure and locate the AWE lesion before and during ablation (Figure 1). In total, 27 patients had a single injury, one patient had 2 lesions, and the other remaining patients had 3 lesions. Ultrasound images showed that there was no pattern in the shapes of the AWE lesions, indicating that the AWE lesions had spiking edges and hypoechoic signals were observed inside the lesions (homogeneous or heterogeneous). In 19 lesions, short or spotted rod-like blood flow signals around or inside the tumor were detected. Meanwhile, low-velocity, high-impedance arterial blood flow was detected. For 13 lesions, no rod-like signal was observed around or inside the lesions.

### Comparison of Abdominal Wall Endometriosis Between Magnetic Resonance Imaging and Color Doppler Ultrasound Before High-Intensity Focused Ultrasound Treatment

This study showed that the observed size of the lesions differed between the two methods (ultrasound and MRI), and the differences between both groups were statistically significant (*P* < 0.05) (Table 3).

**TABLE 3:** Comparison of AWE lesion size between MRI and color Doppler ultrasound before HIFU treatment.

Project	Ultrasound	MRI	*P* value
Maximum radius of lesion (mm)	22.0 (16.3–30.8)	25.0 (21.0–36.5)	0.003
The volume of lesion (mm^3^)	2727.7 (1140.8–6937.4)	4317.2 (2063.9–11,538.8)	0.000

**FIGURE 2:**
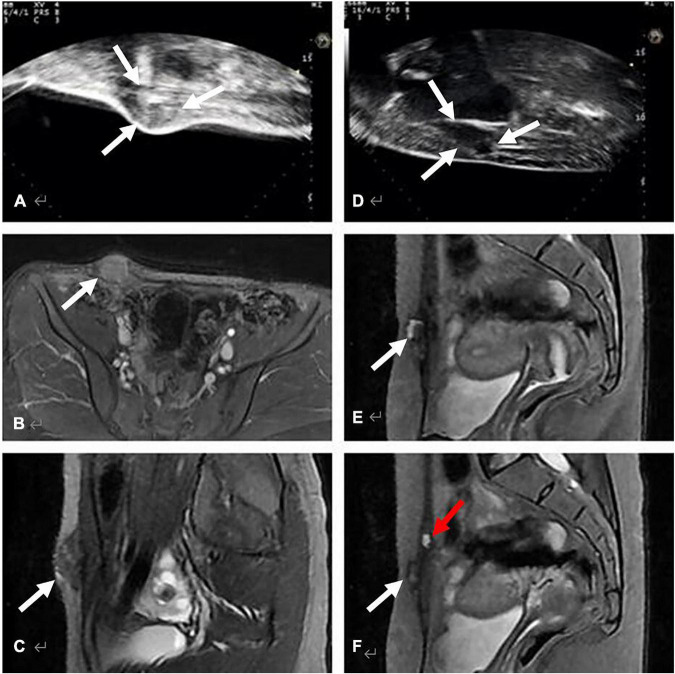
Comparison of AWE lesions between MRI and color Doppler ultrasound before HIFU treatment. **(A–C)** show an AWE lesion on the right horn of the scar, located in subcutaneous fat, invaded the skin and the anterior sheath of the right rectus abdominis: **(A)** Long-axis sonogram showed a hypoechoic, nodular mass (white arrow). **(B,C)** show the axial and sagittal T2-weighted MRI of the same lesion, MRI images were clearer than ultrasound images in terms of size and boundary of the AWE lesion. **(D–F)** show AWE in a 27-year-old woman with complaints of cyclical pain symptoms after cesarean section, due to the presence of 5 cm of abdominal wall fat. Ultrasound images **(D)** revealed only one lesion, while MRI clearly revealed two lesions, along with the location and shape of AWE **(E,F)**.

Figure 2 shows the comparison of AWE lesions between MRI and color Doppler ultrasound prior to HIFU treatment. MRI offers a much more direct and clearer image for the detection of AWE than color Doppler ultrasound. MRI can show the location and shape of AWE clearly. MRI is not affected by the thickness of the abdominal fat, but ultrasound is prone to sound attenuation after passing through the thick abdominal fat layer. MRI can easily detect small lesions that are difficult to detect by ultrasound.

### Magnetic Resonance Imaging Before and After High-Intensity Focused Ultrasound Treatment

**FIGURE 3:**
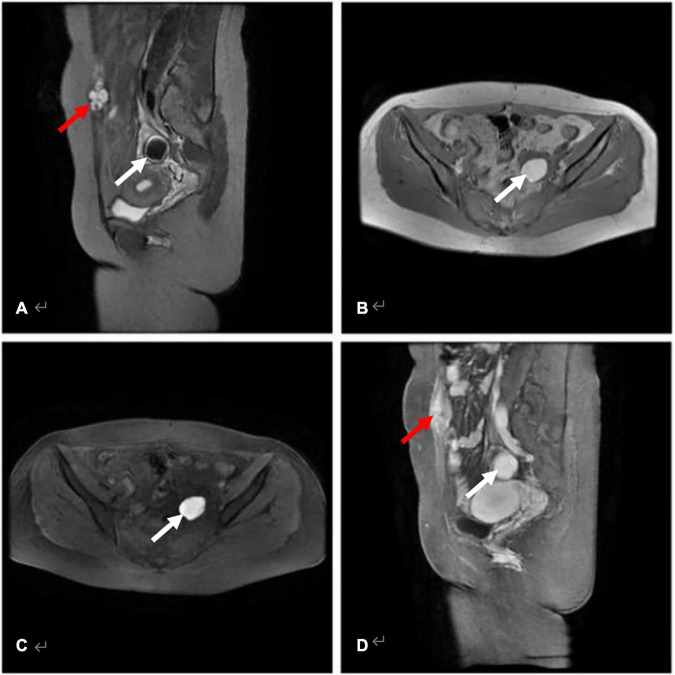
AWE in a 29-year-old woman with a history of transabdominal left ovarian endometriosis cyst excision and cesarean section (median incision). **(A)** Sagittal T2WI with fat-suppression AWE lesion with high signal (red arrow), and cyst of ovarian endometriosis in the attachment area appeared hypointense when compared with muscle (white arrow). **(B,C)** Axial T1WI without and with fat-suppression revealed an endometriosis cyst in the left attachment area with the cyst showing a higher signal than that of muscle (white arrow). **(D)** The AWE lesion (red arrow) and the endometriosis cyst (white arrow) shows evident enhancement on post-contrast T1WI.

Thirty lesions in 29 patients were identified through MRI before HIFU treatment (Note: the number of AWE was determined by MRI findings instead of ultrasound data) (Table 4). They presented mainly (28/29) as a single AWE lesion, which could be located in any layer of the abdominal wall with mixed, isointense or slightly hypo-intense signals on T1WI and hypo-mixed signals (26/30) on T2WI. While on contrast-enhanced MRI, 25 lesions showed significantly enhanced signals. Except AWE, MRI images suggested that 3.4% (1/29) patients had a consolidated uterine leiomyoma, 17.2% (5/29) patients had an endometriosis cyst in the adnexal area, and 24.1% (7/29) patients had consolidated adenomyosis (Table 4). Figure 3 shows the MRI images of AWE and endometriosis cyst in a 29-year-old woman before HIFU treatment.

**TABLE 4:** MRI characteristics before and after HIFU treatment.

Category	Before		After	
	Number of cases	Percentage	Number of cases	Percentage
**Number of lesions**	29		26	
–Single lesion	28	96.6%	25	96.2%
–Multiple lesions (2 lesions)	1	3.4%	1	3.8%
**The location of lesions**				
–In the subcutaneous fat	5	14.3%	4	14.8%
–Invaded anterior layer of sheath of rectus abdominis	15	50%	14	51.9%
–Invaded posterior layer of sheath of rectus abdominis	10	36.7%	9	33.3%
**The plain scan signals**				
**T1WI**				
–Mixed signals	16	53.3%	2	7.4%
–Homogeneous signals	14	46.7%	25	92.6%
**T2WI**				
–Mixed signals	26	86.7%	21	77.8%
–Homogeneous signals	4	13.3%	6	22.2%
**The enhancement signals**				
–Significantly enhanced	25	83.3%	2	7.4%
–Dishomogeneously enhanced	3	10%	0	0
–Annular enhanced	2	2.7%	25	92.6%
**Comorbidities**				
–Adenomyosis	7	24.1%	–	–
–Cystic mass in adnexal area	5	17.2%	–	–
–Uterine leiomyoma	1	3.4%	–	–

**FIGURE 4:**
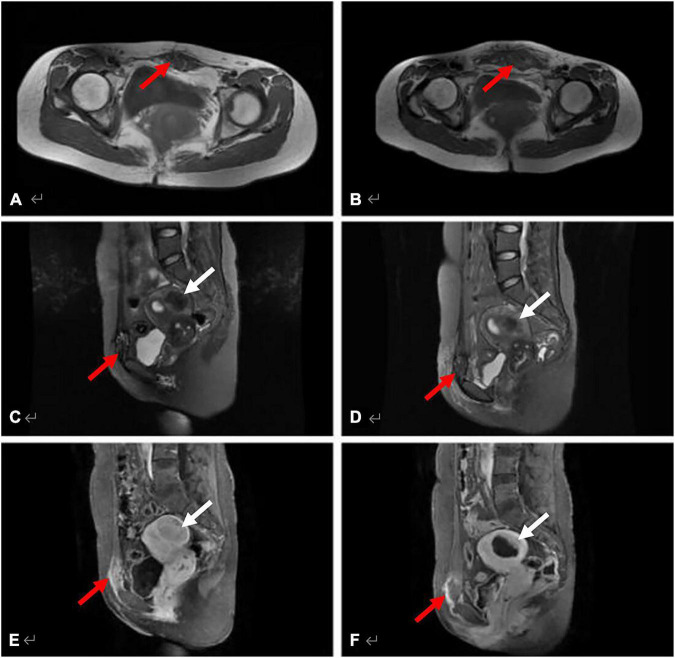
MRI images of AWE in a 42-year-old woman before and after HIFU treatment. The AWE lesion located in the ventral rectus abdominis, invading the rectus sheath. **(A)** Axial T1WI shows the lesion appeared as mixed signal (red arrow) pre-operatively. **(B)** Axial T1WI shows the same lesion appeared slightly hyperintense post-HIFU ablation (red arrow). **(C,D)** Sagittal T2WI with fat-suppression AWE lesion in the rectus abdominis shows high signal (red arrow), and the hysteromyoma in the posterior wall of the uterus appeared hypo-intense as compared with muscle (white arrow). **(E)** The AWE lesion (red arrow) and the hysteromyoma (white arrow) show evident enhancement and slight enhancement, respectively, on post-contrast T1WI. **(F)** Both the AWE lesion (red arrow) and the hysteromyoma (white arrow) clearly showed the non-enhancing areas of post-treatment.

After HIFU treatment, 26 patients with 27 lesions underwent MRI examination, 92.6% (25/27) presented with hyper-intense or slightly hyper-intense signals on T1WI, and 77.8% (21/27) presented with hypo-intense or high-low-mixing signals on T2WI. While on the contrast-enhanced MRI, 92.6% (25/27) of the lesions showed annular enhancement and the absence of contrast agent perfusion in the middle of the lesions. Figure 4 shows the MRI images of AWE in a 42-year-old woman before and after HIFU treatment.

### Calculation of Apparent Diffusion Coefficient Values and the Ablation Rate of the Lesions

**FIGURE 5:**
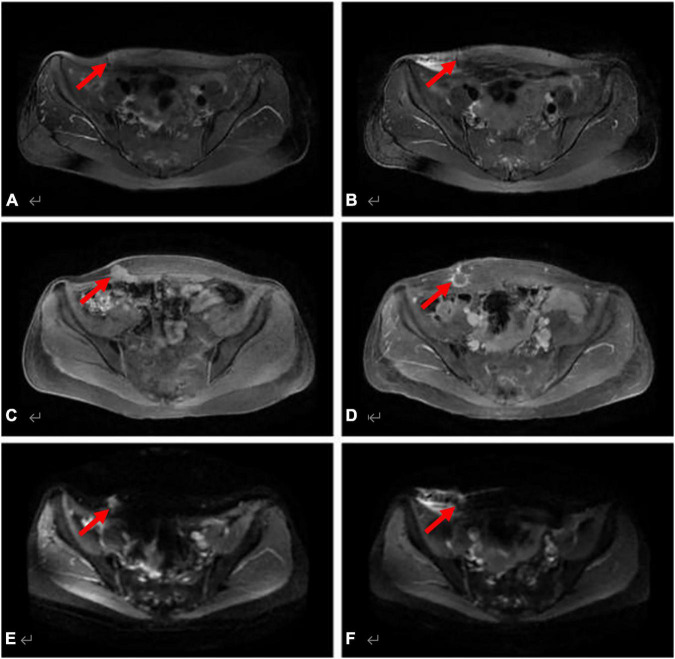
DWI sequence for a 35-year-old patient before and after HIFU treatment. The AWE lesion located at the right corner site of the surgical scar. This chart shows a comparison between pre- and post-treatment. **(A)** Axial T2WI with fat-suppression shows that the lesion appeared slightly hyper-intense (red arrow) pre-operatively. **(B)** Axial T2WI shows the same lesion (red arrow) appeared as signal reduction post-ablation, and the high signal in the surrounding tissue represents edema. **(C,D)** Axial contrast imaging showed evident enhancement pre-treatment (red arrow) and revealed non-enhancing areas of post-treatment (red arrow). **(E,F)** On DWI, the lesion showed high signal pre-operatively, the ADC value was 1.7 × 10^–3^mm^2^/s (*b* = 600 s/mm^2^), which showed slightly low signal post-treatment, and the ADC value was 2.7 × 10^–3^mm^2^/s (*b* = 600 s/mm^2^).

The ADC values of the lesion were statistically significantly higher after HIFU treatment than before (Table 5). The ablation rate of the lesions ranged from 30.0 to 318% with an average of 95.7 ± 1.16%. The majority of patients (20/27, 74.1%) had an ablation rate of more than 50% (Table 6). Figure 5 shows the MRI images that include the DWI sequence obtained from a 35-year-old patient before and after HIFU treatment.

**TABLE 5:** Comparison of ADC values before and after HIFU treatment.

Category	Before HIFU	After HIFU	*P* value
ADC value (×10^–3^mm^2^/s)	1.47 (1.20–1.59)	1.86 (1.61–2.12)	0.005

*Session rates and recurrence rates are presented in Tables 7 and 8, respectively.*

**TABLE 6:** The ablation rate of the lesions on MRI.

No. of lesion	Volume of lesion (mm^3^)	Volume of non-perfusion (mm^3^)	Ablation rate (%)
1*	4767.3	0	0
2*	1628.0	0	0
3	37883.0	6126.8	16.2
4	16156.7	4861.7	30.1
5	1930.8	616.5	31.9
6	4383.2	1888.7	43.1
7	10580.9	4952.1	46.8
8	4095.8	2052.8	50.1
9	15480.9	7841.4	50.7
10	9773.2	5488.1	56.2
11	12542.1	7434.9	59.3
12	3744.9	2244.7	59.9
13	10788.2	6608	61.3
14	2284.5	1536.7	67.3
15	5240.2	3890.4	74.2
16	45228.9	37365.8	82.6
17	58113.6	49363.5	84.9
18	808.8	773.6	95.7
19	22417.2	22252.2	99.3
20	1734.3	1886.5	108.8
21	1051.4	1247	118.6
22	1583.4	1925.6	121.6
23	4978.8	7630.6	153.3
24	2099.1	3670.4	174.9
25	3661.8	9305.9	254.1
26	5395.4	15942.2	295.5
27	2926.2	9312.2	318.2

1*and 2*denote that the two lesions were not ablated at all.

### Remission of Clinical Symptoms, Recurrence, and Ablation Rates of the Lesions in Follow-Up Following High-Intensity Focused Ultrasound Treatment

Patients with ablation rates greater than 50% achieved a higher complete/partial remission rate and had a lower recurrence rate than those with ablation rates less than 50%. The differences between both groups were significant (*P* < 0.05) (Tables 7, 8).

**TABLE 7:** Ablation rates and clinical symptoms remission in 27 lesions.

Ablation rate	Cases	Clinical symptoms	
		Remission (complete/partial)	No remission
0	2	0 (0/0)	2
<50%	5	4 (1/3)	1
≥50%	20	18 (13/5)	2

*Fisher’s exact analysis: p < 0.05.*

**TABLE 8:** Ablation rates and clinical symptom recurrence in 25 lesions.

Project	Cases	Clinical symptom recurrence	
		Yes	No
Ablation rate			
<50%	5	4	1
≥50%	20	4	16

*Fisher’s exact analysis: p < 0.05.*

## Discussion

The main complaint associated with AWE is a mass and period pain in the scar area. Typical clinical manifestations include a history of abdominal surgery, especially a cesarean section. In our study, all patients had a history of cesarean section and 96.6% (28/29) attended the clinic with similar symptoms, which is consistent with previous literature reports (Horton et al., 2008; Ecker et al., 2014; Matalliotakis et al., 2020), but one patient (3.4%) had no specific symptoms and just presented with an abdominal wall mass.

Ultrasound is one of the best methods to detect AWE, but has limited accuracy. This study showed that the observed sizes of lesions differed significantly from ultrasound and MRI scans, and both groups had significant differences (*P* < 0.05). The boundary between the AWE and the surrounding tissue is not clear on ultrasound images. Fat, muscle, fascia, and peritoneum are difficult to visualize by ultrasound; whereas, the better resolution of MRI offers clearer images of the AWE and surrounding features. Furthermore, because pressure is typically applied at the site during the ultrasound imaging process, the shape of the AWE can be compressed.

Magnetic resonance imaging offers a much more direct and clearer image for AWE detection. An MRI can not only clearly show the location and shape of an AWE but also defines its different components by analyzing the signal features. The stages of hemorrhage determine the imaging results. During the first acute period of hemorrhage, the lesion shows a mixed signal on the T2WI STIR sequence (a black ring on the edge of the mass). The hyperintense signal represents the lesion tissue, while the hypointense signal represents a hemorrhage. In this study, most of the lesions in T2WI showed a mixed signal. On T1WI, the majority showed isointense or slightly high signals. Therefore, the mixed signals of the T2WI STIR sequence and the high signals of the T1WI imaging may have great value in detecting AWE.

Enhancement rates in this study are consistent with those reported in previous studies (Busard et al., 2010; Solak et al., 2013). On the enhanced MRI, the contrast of the lesions was significantly enhanced, and the lesion boundaries were relatively more distinct. The depth of the lesions invading the surrounding tissues were revealed by enhanced MRI, which may help to confirm pre-operative localization and allow a surgical intervention. MRI could detect small hemorrhagic lesions and could distinguish between a cystic lesion or solid lesion in patients with AWE. Previous studies have shown that the ablation rate of patients with AWE ranged from 13 to 25% (Ray et al., 2003), which is consistent with our results. At the same time, as a result of MRI findings, a rare case of AWE malignancy was identified by MRI pre-operatively, and the patient was diagnosed with moderately differentiated adenocarcinoma on the post-operative pathological examination. Hence, MRI achieves additional practical values in the differential diagnosis of adenomyosis compared to ultrasound. Post-operative outcomes revealed that the absence of contrast perfusion on MRI can be clinically interpreted as indicative of a lesion transforming into a coagulated necrotic region (Zhang et al., 2014). Our hypothesis is that after HIFU ablation, the tissues became necrotic due to thrombosis of small vessels or capillaries in the lesions and irreversible damage to vascular endothelial cells. The resulting tissue became ischemic, which resulted in the formation of an edematous zone with a clear boundary between the necrotic and surrounding tissue. Diffusion-weighted imaging (DWI) can offer some insight into whether a patient has a benign or malignant tumor. Given the limitations on how much water can spread in the context of a growing malignant tumor, the DWI signal increases and the diffusion of ADC is limited (Genç et al., 2014). To our knowledge, no study has yet characterized the ADC values of AWE with malignant transformations. In this study, the ADC value of AWE lesions increased after HIFU treatment. This can be explained by various factors, such as tissue edema, which may have resulted in an increase in intercellular space and an unlimited area for water molecules to move after HIFU treatment. Edema affects the motion of water molecules more strongly than the coagulative necrosis of tissues, which limits the diffusion of water molecules. Further research is needed to better define the role of DWI in diagnosing AWE and guiding the direction of AWE treatment.

Twenty AWE lesions treated with HIFU had an ablation rate of more than 50%; in addition, eight lesions achieved an ablation rate of over 100%. Patients with an ablation rate greater than 100%, especially those who achieved a maximum ablation rate of 318%, were treated similarly to expanded resection of lesions in traditional surgery (Horton et al., 2008). The results showed that the group with the higher ablation rate (above 50%) had a significantly higher rate of complete/partial remission and a lower recurrence rate than the group with the lower ablation rate (below 50%). This implies that the higher the ablation rate, the better the prognosis in follow-up after HIFU treatment. The classification based on ablation rates of 50% appears to be more reliable than that of 75–80% in USg-HIFU treatment of AWE. AWE was considered to occur mainly on surgical scars, as scar tissue was less vascular and more fibrotic than normal tissue, and the presence of scars may limit access to the target area (Duc and Keserci, 2018). Therefore, the ablation rates following USg-HIFU treatment of AWE were less than those of uterine fibroids. There were five patients with a lower ablation rate of less than 50%, among which four showed a homogeneous intense signal (iso-intense/slightly hyper-intense signal) and one showed a mixed intense signal on T2WI. According to previous studies, uterine fibroids with different SI or hyperintensity on T2WI have different biological characteristics that result in different HIFU or even failed treatment (Pron, 2015; Duc and Keserci, 2018). Furthermore, in this study, two lesions were not ablated with HIFU. These lesions were located in the anterior sheath of the rectus muscle and subcutaneous fat, and measured 17 × 15 × 12 mm and 23 × 16 × 14 mm, respectively. This was likely because the lesions were too small to be identified by intraoperative real-time ultrasound that had lower soft tissue resolution ability than that of MRI. Thus, with MRI examination, the ablation rate of AWE using USg-HIFU treatment could be detected and at least some prediction could be made about remission and recurrence.

The retrospective design and the small sample size are some of the limitations of this study. Due to differences in the performance of the procedures between providers, subtle differences in results were inevitable.

## Conclusion

Magnetic resonance imaging is a practical tool to demonstrate the location, size, and concurrent changes in AWE before and after USg-HIFU treatment and, to a certain extent, can predict disease remission and recurrence.

## Data Availability Statement

The original contributions presented in the study are included in the article/supplementary material, further inquiries can be directed to the corresponding author.

## Ethics Statement

The studies involving human participants were reviewed and approved by the institutional review board. The patients/participants provided their written informed consent to participate in this study. Written informed consent was obtained from the individual(s) for the publication of any potentially identifiable images or data included in this article.

## Author Contributions

SH: conceptualization, methodology, software, investigation, formal analysis, and writing—original draft. YL: data curation, formal analysis, investigation, and visualization. RC: resources and investigation. ZX: conceptualization, funding acquisition, resources, supervision, and writing—review and editing. All authors contributed to the article and approved the submitted version.

## Conflict of Interest

The authors declare that the research was conducted in the absence of any commercial or financial relationships that could be construed as a potential conflict of interest.

## Publisher’s Note

All claims expressed in this article are solely those of the authors and do not necessarily represent those of their affiliated organizations, or those of the publisher, the editors and the reviewers. Any product that may be evaluated in this article, or claim that may be made by its manufacturer, is not guaranteed or endorsed by the publisher.
